# Caffeine increases striatal dopamine D_2_/D_3_ receptor availability in the human brain

**DOI:** 10.1038/tp.2015.46

**Published:** 2015-04-14

**Authors:** N D Volkow, G-J Wang, J Logan, D Alexoff, J S Fowler, P K Thanos, C Wong, V Casado, S Ferre, D Tomasi

**Affiliations:** 1 Intramural Research Program, National Institute on Alcohol Abuse and Alcoholism, Bethesda, MD, USA; 2 Brookhaven National Laboratory, Upton, NY, USA; 3 Department of Biochemistry and Molecular Biology, University of Barcelona, Barcelona, Spain; 4 Intramural Research Program, National Institute on Drug Abuse, Baltimore, MD, USA

## Abstract

Caffeine, the most widely consumed psychoactive substance in the world, is used to promote wakefulness and enhance alertness. Like other wake-promoting drugs (stimulants and modafinil), caffeine enhances dopamine (DA) signaling in the brain, which it does predominantly by antagonizing adenosine A_2A_ receptors (A_2A_R). However, it is unclear if caffeine, at the doses consumed by humans, increases DA release or whether it modulates the functions of postsynaptic DA receptors through its interaction with adenosine receptors, which modulate them. We used positron emission tomography and [^11^C]raclopride (DA D_2_/D_3_ receptor radioligand sensitive to endogenous DA) to assess if caffeine increased DA release in striatum in 20 healthy controls. Caffeine (300 mg p.o.) significantly increased the availability of D_2_/D_3_ receptors in putamen and ventral striatum, but not in caudate, when compared with placebo. In addition, caffeine-induced increases in D_2_/D_3_ receptor availability in the ventral striatum were associated with caffeine-induced increases in alertness. Our findings indicate that in the human brain, caffeine, at doses typically consumed, increases the availability of DA D_2_/D_3_ receptors, which indicates that caffeine does not increase DA in the striatum for this would have decreased D_2_/D_3_ receptor availability. Instead, we interpret our findings to reflect an increase in D_2_/D_3_ receptor levels in striatum with caffeine (or changes in affinity). The association between increases in D_2_/D_3_ receptor availability in ventral striatum and alertness suggests that caffeine might enhance arousal, in part, by upregulating D_2_/D_3_ receptors.

## Introduction

Caffeine is the most widely consumed psychoactive substance.^1^ Its behavioral arousing pharmacological effects are similar to those of stimulant drugs (amphetamine and methylphenidate) and modafinil, which are drugs that increase dopamine (DA) signaling by blocking DA transporters and/or enhancing DA release from the terminals.^2, 3, 4^ The DA-enhancing effects of these drugs underlie their arousing^5, 6^ and reinforcing effects.^7, 8, 9, 10^ In contrast, preclinical studies indicate that caffeine's pharmacological effects are mediated by its antagonism of adenosine receptors (A_1_ and A_2A_ subtypes).^11^ In particular, its antagonism of A_2A_ receptors (A_2A_R) in striatum has been implicated in its dopaminergic effects.^12^ Similarly, caffeine-induced increases in locomotor activity^13^ and arousal^14^ appear to be mediated by A_2A_R as they are absent in A_2A_R knockout mice, and silencing the expression of A_2A_R with short-hairpin RNA in the nucleus accumbens interferes with caffeine's effects on wakefulness.^15^

The striatum expresses high levels of A_2A_R where they are co-expressed with postsynaptic D_2_ receptors (D_2_R) forming A_2A_R-D_2_R heteromers.^16, 17, 18^ Through allosteric and second-messenger interactions adenosine inhibits D_2_R signaling. Thus, in striatal neurons, A_2A_R agonists decrease D_2_R agonist binding.^19^ Caffeine, by blocking A_2A_R, could enhance DA signaling through the unopposed D_2_R.^20^ Though it was initially postulated that caffeine antagonism of adenosine A_1_ receptors resulted in DA increases in the nucleus accumbens,^21^ this finding was only obtained after very high doses of caffeine and was not corroborated by others.^22, 23^ Furthermore, a brain imaging study with [^11^C]raclopride, which is a radioligand that competes with endogenous DA for binding to D_2_ and D_3_ receptors (D_2_/D_3_R), showed that oral caffeine (200 mg) increased its binding in striatum,^24^ which is inconsistent with DA increases. However, the small sample size from the study (*n*=8) precludes its generalizability. Thus, the question of whether caffeine increases striatal DA and the mechanism(s) of action for caffeine's alerting effects in the human brain remain unclear.

To assess whether caffeine increases DA in the human brain, we used positron emission tomography (PET) and [^11^C]raclopride^25^ and tested 20 healthy controls once with placebo and once with oral caffeine. A 300-mg dose of caffeine was selected to reflect the average amount of caffeine in 2–3 cups of coffee. We hypothesized that caffeine would not increase DA in striatum but instead would enhance striatal DA signaling by increasing D_2_R.

## Materials and methods

### Subjects

This study included 20 healthy male controls (38±8 years of age, body mass index 26±3; years of education 14±2) recruited through advertisements in local newspapers. Exclusion criteria included consumption of more than two caffeine beverages per day, current or past psychiatric disease as per DSM IV including any substance use disorder (smokers were excluded); past or present history of neurological, cardiovascular or endocrinological disease; history of head trauma with loss of consciousness greater than 30 min; and current medical illness. Seventeen of the participants reported that they did not drink coffee (or caffeinated beverages), one reported one cup a day and two reported two cups a day. Written informed consent was obtained from all the subjects and the studies were reviewed and approved by the Institutional Review Board at Stony Brook University Medical Center.

### Self-reports and scales and cardiovascular measures

To study the behavioral effects of caffeine, we assessed self-reports for subjective perception of ‘alertness', ‘tiredness', ‘sleepiness' and ‘mood' using analog scales (rated from 1 to 10) that were obtained before and at 30 and 120 min after placebo or caffeine administration, as previously described.^26^ The use of analog scales to assess self-reports of drug effects have been shown to be reproducible and to predict drug responses.^27^ For the correlation analysis, we used the measures obtained 120 min after caffeine administration (at the end of the [^11^C]raclopride scan), which is within the time for peak caffeine effects (60–120 min).^28^

Heart rate and blood pressure were recorded three times at five-minute intervals before the administration of placebo or caffeine and periodically thereafter until 120 min post placebo or post caffeine. The measures taken before placebo or caffeine were averaged (pre-drug measures) and those taken 60–120 min post administration were averaged as post-drug measures. Effects of the drug were evaluated as paired *t*-test comparisons between the pre- and the post-drug measures.

### Measures of caffeine in plasma

Venous blood was drawn before and at 30, 60 and 120 min after caffeine administration. Caffeine in plasma was quantified using high-performance liquid chromatography.^29^

### PET scan

We used an HR+ tomography (resolution 4.5 × 4.5 × 4.5 mm full width at half maximum, 63 slices) with [^11^C]raclopride 4–8 mCi (specific activity 0.5–1.5 Ci μM^−1^ at the end of bombardment). The procedures for imaging were as previously described.^30^ Briefly, 20 dynamic emission scans were obtained immediately after injection for a total of 54 min. The participants were scanned with [^11^C]raclopride twice, once with placebo and once with caffeine; the placebo scans were done 2 h before the caffeine scan. Caffeine (300 mg) and placebo (sugar tablet) were administered orally 60 min before the [^11^C]raclopride injection. We chose 60 min as peak effects from oral caffeine occur at ~60 min when it is administered as a tablet.^28^ The half life of caffeine in plasma is ~3–5 h,^31^ so this time point ensured high plasma caffeine levels during the PET measurements (60–120 min post caffeine).

### PET image analysis

We analyzed the nondisplaceable binding potential (BP_ND_) images using Statistical Parametric Mapping (SPM8; Wellcome Trust Centre for Neuroimaging, London, UK), which enabled us to make comparisons on a pixel-by-pixel basis.^32^ Specifically, we estimated for each voxel the distribution volume ratio, which corresponds to the equilibrium measurement of the ratio of the radiotracer's tissue concentration in the striatum to that in the cerebellum, which is used as a reference region.^33^ These images were then spatially normalized to the stereotactic space of the Montreal Neurological Institute using a 12-parameter affine transformation and 2-mm isotropic voxels. A custom Montreal Neurological Institute template, which was previously developed using images from 34 healthy subjects acquired with [^11^C]raclopride and the same PET scanning sequence,^34^ was used for the spatial normalization of the distribution volume ratio images. The voxels of the distribution volume ratio images correspond to BP_ND_ +1.

An independent region-of-interest (ROI) analysis was performed using preselected ROIs in caudate, putamen and ventral striatum (VS) as previously described^25^ to corroborate the SPM findings. The ROI measures were used for the correlation analysis with the behavioral measures that were significantly affected by caffeine and to assess the correlations with the levels of caffeine in plasma.

### Statistical analyses

The brain maps (BP_ND_) were spatially smoothed in SPM8 using an 8-mm isotropic Gaussian kernel to minimize the effects associated with the variability of the brain anatomy across subjects. A striatal mask (dorsal striatum and VS) was created using the digital anatomical brain atlases provided with the MRIcro software (www.cabiatl.com/mricro/). Specifically, the voxels corresponding to striatum (caudate, putamen and VS) were defined in the Montreal Neurological Institute stereotactic space using the Automated Anatomical Labeling atlas.^35^ One-way (within-subjects) analysis of variance was used to assess drug effects (placebo vs caffeine) on BP_ND_ with SPM8. Statistical significance was set by the stringent threshold *P*_FWE_<0.05, corrected for multiple comparisons at the voxel level (within a striatal mask) using the random field theory with a family-wise error correction. For visualization purposes regarding the MRI location of the regions that differed significantly between placebo and caffeine, we used an uncorrected threshold of *P*<0.01.

For the independent ROI analysis, statistical significance was set at *P*<0.05, if it corroborated the SPM findings.

For the behavioral and cardiovascular measures, we compared each time point between the placebo and caffeine scores using repeated analysis of variance. Correlation analyses were done to assess the relationship between the regions where caffeine changed BP_ND_ and the behavioral measures that were significantly affected by caffeine. Significance was set at *P* <0.05.

## Results

### Effects of caffeine on self-reports and on cardiovascular measures

**Figure 1:**
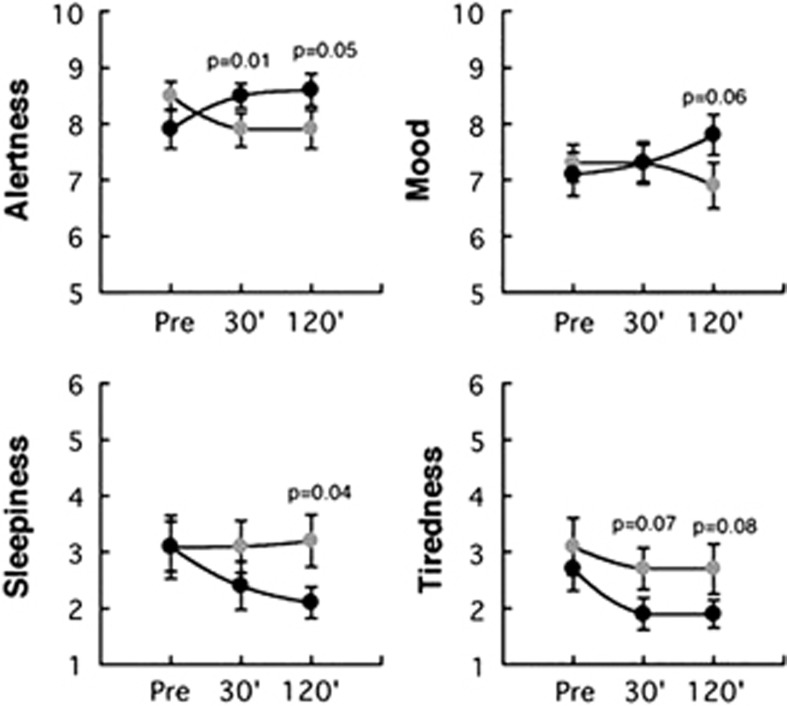
Behavioral effects of placebo and caffeine before and 30 and 120 min after their administration. Significance corresponds to comparison between placebo (gray symbols) and caffeine (black symbols) and values correspond to means and standard errors.

Comparisons between caffeine and placebo for the corresponding time measures showed significantly higher self-reports of ‘alertness' both at 30' (*P*=0.05) and at 120' (*P*=0.01) and lower scores in ‘sleepiness' at 120' (*P*=0.04) than placebo. Differences between caffeine and placebo for scores on mood and tiredness only reached trend effects (*P*>0.06<0.09; Figure 1).

The average cardiovascular measures were not significantly affected by caffeine (pre vs post). Specifically, for heart rate, pre vs post placebo (70±10 vs 64±9) or pre vs post caffeine (66±9 vs 65±11); for systolic pressure, pre vs post placebo (124±6 vs 122±7) or pre vs post caffeine (128±11 vs 129±9); or for diastolic pressure, pre vs post placebo (67±10 vs 65±9) or pre vs post caffeine measures (71±12 vs 69±11); none of which differ significantly from one another.

### Measures of caffeine in plasma

There were no detectable levels of caffeine on the plasma samples taken before caffeine administration. Measures of caffeine concentration in plasma were 4.7±2 μg ml^−1^ at 30 min, 5.2±1 μg ml^−1^ at 60 min and 4.8±0.6 μg ml^−1^ at 120 min. This corroborated that we had peak levels of caffeine in plasma at the time of [^11^C]raclopride injection (60 min post caffeine) and high levels at the time of the behavioral measures (30 and 120 min post caffeine).

### Effects of caffeine on D_2_/D_3_R availability

**Figure 2:**
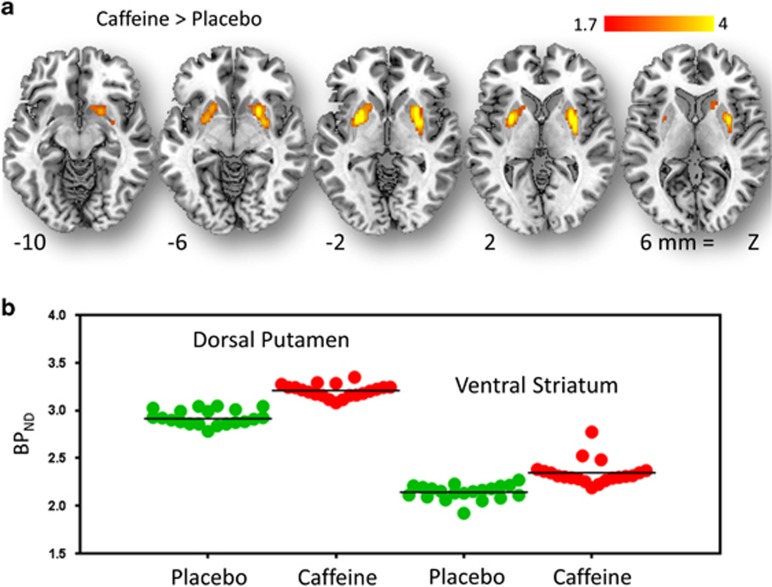
(**a**) Brain maps obtained with Statistical Parametric Mapping (SPM) showing significant differences in D_2_/D_3_R availability, which was quantified as nondisplaceable binding potential (BP_ND_), between placebo and caffeine for the contrast caffeine >placebo. Threshold for significance corresponds to *P*_u_<0.01, clusters >100 voxels. (**b**) Individual values for BP_ND_ from measures extracted in dorsal putamen and in ventral striatum after placebo and after caffeine.

SPM revealed that caffeine increased D_2_/D_3_R availability (observed as increases in BP_ND_) in right and left striatum (including dorsal putamen and VS) as shown both by the averaged statistical maps as well as the individual values extracted from the center of the significant clusters (Figure 2, Table 1).

The independent ROI analyses, corroborated that caffeine when compared with placebo induced small but significant increases in BP_ND_, in putamen (placebo: 2.84±0.37 vs caffeine: 2.97±0.35; *P*=0.05) and in VS (placebo: 2.69±0.31 vs caffeine: 2.84±0.39, *P*=0.05) but not in caudate.

### Correlations between caffeine-induced changes in D_2_/D_3_R availability and behavior and plasma levels

The correlation analysis with the striatal ROI and the behavioral measures showed a significant positive correlation between VS and alertness (*r*=0.56, *P*=0.01) such that increases in D_2_/D_3_R availability with caffeine were associated with increases in alertness.

The correlation analysis between caffeine-induced changes in D_2_/D_3_R availability in striatum and levels of caffeine in plasma were not significant.

## Discussion

Here we show that caffeine increases D_2_/D_3_R availability in striatum (evidenced as increases in BP_ND_ in dorsal putamen and VS) in a group of healthy controls with low levels of daily caffeine intake. These findings are consistent with findings from a prior PET [^11^C]raclopride study done in a small group of subjects (eight habitual coffee drinkers) that also reported increases in D_2_/D_3_R availability in striatum with caffeine (200 mg).^24^ The findings from these two studies thus suggest that caffeine at doses typically consumed by humans might enhance DA signaling by increasing D_2_/D_3_R levels or their affinity rather than by increasing DA release in the striatum.

Here we interpret our results of increases in BP_ND_ (in BP_ND_ availability) with caffeine to suggest that they reflect increases in D_2_/D_3_R levels rather than reflecting decreases in endogenous DA, which is the way that typically increases in BP_ND_ are interpreted (reduced competition from DA to bind to D_2_/D_3_R). The reasons for this interpretation follow. First, it is recognized that alerting drugs (amphetamine, methylphenidate and modafinil) increase DA release in the striatum.^3, 25, 36^ Second, clinical studies have shown that the DA increases in striatum induced by stimulant drugs are associated with increases in alertness.^5^ Finally, preclinical studies have shown that the increases in striatal DA induced by stimulants and modafinil is necessary for their wake-promoting actions.^6^ Thus, if caffeine had reduced DA in the striatum, this would have resulted in an increase in tiredness and sleepiness instead of the increases in alertness observed after caffeine administration. Our interpretation that the increases in striatal D_2_/D_3_R availability in VS with caffeine reflect an increase in D_2_/D_3_R levels is also consistent with our findings that downregulation of D2/D3R in VS after sleep deprivation is associated with reduced alertness.^5^

Striato-pallidal neurons adjust their excitability by changing D_2_R levels in the membrane.^37^ Thus, D_2_R downregulate with DA stimulation^38^ and upregulate with reduced DA signaling.^39, 40^ DA stimulation of D_2_R triggers their internalization,^38^ which can then be recycled or degraded.^38, 41^ Internalization of D2R is regulated by A_2A_R,^42^ agonists facilitate its internalization through the binding of β-arrestin 2 to A_2A_R-D_2_R receptor heteromers^43^ whereas A_2A_R antagonists interfere with D_2_R internalization in striatal neurons.^44^ Thus, caffeine might interfere with a tonic A_2A_R-dependent internalization of D_2_R mediated by endogenous adenosine, which could contribute to its psychostimulant effects.^14, 19, 45, 46^ Indeed, our findings along with those previously reported showing that caffeine increased D_2_R availability in striatum,^24^ support this interpretation. As caffeine modulates DA signaling, in part, by its antagonism of A_2A_R,^47^ caffeine-induced D_2_R increases in striatum would be consistent with caffeine's antagonism of A_2A_-mediated D_2_R internalization. Indeed, A_2A_ receptor knockout mice show increased D_2_R levels in striatum;^48^ though we cannot necessarily equate the chronic state of a knockout with the effects from acute caffeine exposure.

However, regardless of the mechanism responsible for the increases in striatal D_2_/D_3_R availability, our results indicate that in humans, caffeine at the doses typically consumed, does not increase DA in the striatum. This is consistent with findings from microdialysis studies in rodent showing that caffeine (0.25–5 mg kg^−1^ intravenously or 1.5 to 30 mg kg^−1^ intraperitoneally) did not increase DA in the nucleus accumbens,^22, 23^ though a study reported increases with a large (10 mg kg^−1^ intraperitoneally) but not a lower caffeine dose (3 mg kg^−1^ intraperitoneally).^21^ Thus, on the basis of the current and prior findings^24^ and the preclinical results, caffeine at doses that are relevant to human consumption does not appear to increase DA in the nucleus accumbens. As the ability of drugs of abuse to increase DA is necessary for their rewarding effects and for the neuroadaptations associated with the addiction phenotype,^49^ this could explain why caffeine does not produce the compulsive administration and the loss of control that characterizes addiction.^50^

Caffeine-induced increases in D_2_/D_3_R in VS were associated with increases in alertness. This association between alertness and D_2_/D_3_R replicates our previous findings with sleep deprivation but in the opposite direction, in which we showed that the decreases in D_2_/D_3_R availability in VS with sleep deprivation were associated with reductions in alertness.^5^ In the prior PET study, caffeine-induced increases in striatal D_2_/D_3_R availability were associated with reduced tiredness.^24^ Thus this provides evidence that enhanced signaling through D_2_/D_3_R in striatal regions might enhance alertness or decrease tiredness, whereas reduced signaling might decrease alertness or increase fatigue.

### Study limitations

Traditionally, increases in D_2_/D_3_R availability with [^11^C]raclopride, as observed here, have been interpreted to reflect decreases in DA release. Instead, our model leads us to interpret them as increases in D_2_/D_3_R levels and/or increases in affinity. However, our model cannot rule out the potential confound that more than one factor could be affecting the binding of [^11^C]raclopride. In this respect, preclinical experiments that use more selective compounds should be performed to investigate whether caffeine's effects on [^11^C]raclopride binding reflect changes in the expression or in the affinity of D_2_/D_3_R and whether these effects reflect caffeine's antagonism at A_2A_R. Also because [^11^C]raclopride binds to both D_2_R and D_3_R,^51^ we cannot distinguish whether caffeine-induced increases in striatal BP_ND_ reflects only increases in D_2_R or also in D_3_R. However, in putamen where the relative density of D_3_R is much lower than that of D_2_R,^52^ the effects of caffeine are likely to reflect D_2_R. Another potential confound in our study is that caffeine significantly reduces cerebral blood flow,^53^ which could interfere with the BP_ND_ measures as cerebral blood flow effects differ between cerebellum and striatum.^54^ However, because caffeine decreases cerebral blood flow in striatum to a greater extent than in cerebellum,^54^ this would lead to decreases in striatal BP_ND_, whereas we showed the opposite; that is increases in striatal BP_ND_ with caffeine, indicating that our findings are not due to caffeine-induced changes in cerebral blood flow. Though the raclopride PET method cannot distinguish between presynaptic and postsynaptic D2/D3R, the fact that caffeine is an antagonist at A2A receptors, which are expressed in medium spiny neurons expressing D2R but not in DA neurons lead us to presume that the effects are postsynaptic. Another confound in our studies is the order effect as placebo was always given 2 h before caffeine. However, studies that have evaluated test–retest reproducibility for raclopride binding (including ours)^55, 56^ have reported no significant differences between measures even when the repeated measures were performed on the same day^57^ as per the current study, indicating that the order effect is unlikely to account for our findings. We are unable to assess if the participants were able to determine if they received caffeine or placebo as we did not query them at the end of the study. Finally, we did not collect blood samples for epinephrine and norepinephrine, which are increased by caffeine.^58^ Thus, we cannot rule out the contribution of caffeine's effects in the autonomic system on the behavioral effects of caffeine. Nonetheless, the significant association between increases in D2R availability in VS and alertness indicates that caffeine's effects on D2R signaling contribute to its alerting effects.

## Conclusion

We show a significant increase in D_2_/D_3_R availability in striatum with caffeine administration, which indicates that caffeine at doses consumed by humans does not increase DA in striatum. Instead we interpret our findings to indicate that caffeine's DA-enhancing effects in the human brain are indirect and mediated by an increase in D_2_/D_3_R levels and/or changes in D_2_/D_3_R affinity.

## Figures and Tables

**Table 1:** Statistical significance for changes in BP_ND_ for the contrast caffeine greater than placebo

*Region*	*MNI coordinates (mm)*			k	T	Z
	*x*	*y*	*z*	*Voxels*		
L Striatum Putamen	−22	4	−2	222	5.5	4.9
L Striatum	−16	14	−4		3.5	3.3
R Striatum Putamen	32	0	2	340	5.2	4.7
R Striatum Putamen	28	10	−2		4.4	4.1

Abbreviations: BP_ND_, nondisplaceable binding potential; FWE, family-wise error.

The locations of the clusters are based on the coordinates from the stereotactic space of the Montreal Neurological Institute (MNI) in (x, y, z). The values correspond to the *T*-scores and significance was set at *P*_*FWE*_<0.05.
